# Modulation of Intestinal–Bone Crosstalk by a Standardised Nutraceutical Combination: An In Vitro Mechanistic Study

**DOI:** 10.3390/nu18091331

**Published:** 2026-04-23

**Authors:** Rebecca Galla, Simone Mulè, Francesca Parini, Francesca Uberti

**Affiliations:** 1 Noivita Srls, Spin Off University of Piemonte Orientale, Strada Privata Curti 7, 28100 Novara, Italy; rebecca.galla@noivita.it (R.G.); francescaparini00@gmail.com (F.P.); 2 Department for Sustainable Development and Ecological Transition, University of Piemonte Orientale (UPO), Piazza Sant’Eusebio 5, 13100 Vercelli, Italy; simone.mule@uniupo.it

**Keywords:** gut–bone axis, osteoclastogenesis, functional bioactives, nutraceutical formulation

## Abstract

**Background/Objectives**: Natural multi-component nutraceutical formulations may modulate interconnected pathways involved in metabolic and bone health. This study evaluated, using in vitro models, the effects of a standardized botanical–vitamin formulation on intestinal barrier integrity, osteoblastic activity, and osteoclast differentiation, focusing on intestinal-bone crosstalk, redox-inflammatory signalling, and potential synergistic interactions among components. **Methods**: A combined in vitro approach using intestinal, osteoblastic, and osteoclastic cell models was applied to assess a formulation containing characterized plant extracts and vitamin D_3_. The study evaluated cytocompatibility, intestinal barrier function, cellular uptake, and the modulation of markers related to osteogenesis and osteoclastogenesis, using biochemical, molecular, and enzymatic assays, as well as oxidative stress measurements and synergy analysis. **Results**: The formulation maintained intestinal barrier integrity and bioavailability without cytotoxicity, promoted osteoblastic differentiation and reduced oxidative stress, while inhibiting osteoclast differentiation and resorptive activity. These effects were associated with modulation of inflammatory and redox-related signalling pathways and showed additive to synergistic interactions among components. **Conclusions**: These findings support a multi-target nutraceutical approach that can concurrently influence intestinal barrier and bone remodelling in vitro, offering mechanistic evidence for its role in modulating the gut–bone axis, and highlight the need for further studies in advanced models and clinical trials.

## 1. Introduction

Osteoporosis is a skeletal condition arising from an imbalance in bone turnover, in which increased osteoclast activity is not adequately counterbalanced by osteoblastactivityic function, ultimately leading to reduced bone mineral density (BMD) and weakening of the bone structure [1,2]. Defined by the WHO in 1993 as a progressive systemic skeletal disease marked by low bone density, microstructural deterioration, fragility, and higher fracture risk [3], it can be further assessed using biochemical markers of bone turnover [4,5].

Bone is a dynamic metabolic tissue that plays fundamental roles in the structure of the skeleton, organ protection, and mineral metabolism [6]. The integrity of cancellous bone is dependent on the equilibrium between the processes of formation and resorption. These processes are regulated by a complex interaction between bone cells and molecular signals [7]. The predominant cellular components involved in this process are osteoblasts, which synthesise the extracellular matrix and mineralise bone, and osteoclasts, which are multinucleated cells responsible for bone resorption [8]. Osteocytes, which are derived from osteoblasts, perform a regulatory function by perceiving mechanical stimuli and orchestrating communication between osteoblasts and osteoclasts. This is achieved through paracrine signals and soluble factors [9,10].

Bone remodelling is a cyclical process comprising four phases: resorption, reversal, formation, and quiescence. During resorption, osteoclasts degrade the mineral and organic matrix, releasing calcium and phosphate into the bloodstream [11], while in the formation phase, osteoblasts deposit and mineralise new osteoid. This balance is regulated by endocrine and local factors, particularly the RANK/RANKL/OPG system, which controls osteoclast differentiation and activity, as well as systemic hormones such as PTH, calcitonin, and estrogen [12,13,14]. Cytokines and growth factors, including BMPs, IGF-1, and TGF-β, further modulate osteoblast proliferation and maturation, influencing bone quality and density. Disruption of the resorption–formation balance leads to bone loss and fragility [15]. It is crucial to understand the cellular and molecular mechanisms underlying bone remodelling to develop targeted therapies, such as anti-resorptive and osteoformative drugs, and to correctly interpret bone turnover biomarkers used in clinical practice. Although treatments like hormone replacement therapy and bisphosphonates are widely used, their long-term use is often limited by adverse effects and poor patient compliance, highlighting the need for safer, multi-target natural alternatives [16]. Growing evidence shows that bone homeostasis is closely linked to intestinal homeostasis through the gut–bone axis. The intestine, via the gut microbiota, epithelial barrier, and immune components, regulates systemic inflammation and bone metabolism. Disruptions such as dysbiosis or increased permeability can promote low-grade inflammation, enhance osteoclast activity, and alter the RANKL/OPG axis. These insights support exploring multi-target natural strategies to modulate both gut and bone health [17].

For this reason, several natural compounds and nutrients have been investigated for their potential to modulate osteoblast and osteoclast activity in vitro, thereby influencing bone remodelling and mitigating mechanisms underlying osteoporosis. Among these, *Artemisia capillaris*, *Boswellia serrata*, *Eruca sativa*, and vitamin D3 have shown promising cellular effects.

Bioactive compounds present in *Artemisia capillaris*, particularly phenolic acids and coumarins, have been associated with promoting osteogenic activity in MC3T3-E1 cells, as evidenced by increased cell proliferation and differentiation, along with elevated expression of mineralisation markers, including osterix, alkaline phosphatase (ALP), and osteocalcin [18]. Simultaneously, the extracts inhibit osteoclast formation and activity from bone marrow progenitor cells, downregulating Tartrate-Resistant Acid Phosphatase (TRAP) expression and modulating the RANKL-RANK-OPG signalling pathway. Additionally, the antioxidant properties of the extract activate the Nrf2/HO-1 pathway and reduce intracellular oxidative stress, protecting bone cells from apoptosis [19]. These dual effects support both osteoblast function and inhibition of osteoclast-mediated degradation, contributing to the maintenance of bone mass and integrity [20].

*Boswellia serrata*, particularly its active constituent 3-O-acetyl-11-keto-β-boswellic acid (AKBA), exerts anti-inflammatory and anti-osteoclastogenic effects in vitro. In human mononuclear cell-derived osteoclast cultures, AKBA reduces osteoclast differentiation and activity, decreasing the formation of multinucleated osteoclasts and the expression of genes such as TRAP, cathepsin K, and Nuclear Factor of Activated T Cells 1 (NFATc1) [21]. These effects are mediated by inhibition of the NF-κB pathway, leading to reduced production of pro-inflammatory cytokines, including TNF-α and IL-1β, which normally promote osteoclastogenesis. In parallel, osteoblast studies indicate that Boswellia extracts may enhance matrix synthesis and ALP activity, suggesting that modulation of inflammation indirectly supports bone formation. Further mechanistic studies highlight the involvement of Mitogen-Activated Protein Kinase (MAPK) and Extracellular Signal-Regulated Kinase (ERK) signalling in regulating osteoblast proliferation and differentiation [22].

Emerging evidence suggests that *Eruca sativa* (*E. sativa*) possesses significant potential to modulate bone cell environments. Leaf extracts, rich in flavonoids and glucosinolates, have been shown in osteoblast cultures to reduce reactive oxygen species and modulate pro-inflammatory cytokine expression, including IL-6 and TNF-α [23,24,25]. These actions create a cellular environment conducive to osteoblast survival and differentiation, potentially enhancing matrix mineralisation. While direct modulation of osteoclasts and RANKL signalling has not been fully explored, the anti-inflammatory and antioxidant properties of *E. sativa* suggest a supportive role in preserving bone mass and reducing osteoclast-mediated bone loss [26,27].

Vitamin D3 (colecalciferol) regulates bone physiology by acting on both osteoblasts and osteoclasts. It promotes osteoblast differentiation and mineralisation by increasing ALP, osteocalcin, and osteopontin expression [28], and modulates osteoclast formation and activity through the RANKL/OPG axis. Additionally, it supports intracellular calcium metabolism and reduces apoptosis under stress [29]. These actions highlight its key role in maintaining bone homeostasis and preventing osteoporotic bone loss [30].

Based on this evidence, the present study investigated the biological effects of a nutraceutical formulation (Mix) containing *Artemisia capillaris*, *Eruca sativa*, *Boswellia serrata*, and vitamin D3. The combination of these agents is hypothesised to provide a synergistic effect, targeting multiple signalling pathways, including inflammatory cytokines and oxidative stress, more effectively than any single component alone. To replicate key features of osteoporotic conditions, such as reduced osteoblastic differentiation, increased oxidative stress, and enhanced osteoclastogenesis, we employed an in vitro model of bone loss including osteoblast and osteoclast cultures. The study aimed to determine whether Mix could decrease osteoclast activity, restore osteoblastic function, and alter pertinent molecular pathways, including oxidative stress signalling, NF-κB, and RANKL/OPG. These results suggest a possible mechanistic basis for its application in bone health.

## 2. Materials and Methods

### 2.1. Natural Extract Characteristics

#### 2.1.1. Determination of Boswellic Acids Content in *Boswellia serrata* Extract

The content of boswellic acids in *Boswellia serrata* extract was evaluated by high-performance liquid chromatography (HPLC; Agilent, Santa Clara, CA, USA) following previously described methodologies [31]. The hydroalcoholic extract (10:1) was accurately weighed and dissolved in methanol to prepare a working solution at 1 mg/mL. Prior to injection, the sample was sonicated for 15 min at room temperature and then filtered through a 0.45 μm PTFE membrane.

Chromatographic analysis was carried out with UV detection at 210 nm, a wavelength appropriate for detecting boswellic acid derivatives. Total organic acids were quantified by integrating the chromatographic peaks corresponding to the main boswellic acid components and expressing their cumulative content as a percentage (*w*/*w*) relative to the dry extract.

Individual compounds, including 3-O-acetyl-11-keto-β-boswellic acid (AKBA), acetyl-β-boswellic acid (AβBA), and acetyl-α-boswellic acid (AαBA), were identified by matching retention times and UV spectral profiles with those of certified reference standards. Quantitative determination was performed using external calibration curves generated over the concentration range of 5–100 μg/mL, which showed satisfactory linearity (R^2^ > 0.995).

#### 2.1.2. Determination of Total Flavonoid Marker Content in *Eruca sativa* Extract

According to previously published analytical procedures [32], high-performance liquid chromatography (HPLC; Agilent, Santa Clara, CA, USA) was employed to estimate the total flavonoid marker content of *Eruca sativa Mill.* leaf extract, expressed as icariin equivalents. The dried extract was precisely weighed and dissolved in methanol to prepare a 1 mg/mL stock solution. The sample was then sonicated for 15 min at room temperature to ensure complete dissolution, and subsequently filtered through a 0.45 μm PTFE membrane before chromatographic analysis. Chromatographic separation was achieved on a reversed-phase C18 column (250 × 4.6 mm, 5 μm) maintained at 30 °C. The mobile phase consisted of water with 0.1% formic acid (solvent A) and acetonitrile (solvent B) under gradient elution at a flow rate of 1.0 mL/min. Detection was performed using a UV–visible detector set at 270 nm.

The total flavonoid-like content was quantified using an external calibration curve with icariin as the reference standard. The calibration range (5–100 μg/mL) showed satisfactory linearity, with correlation coefficients (R^2^) exceeding 0.995.

#### 2.1.3. Determination of Scoparone and Chlorogenic Acid in *Artemisia capillaris* Extract

An HPLC method was used to quantify scoparone and chlorogenic acid in the aqueous extract of *Artemisia capillaris* [33]. To prepare the analytical solution, the dried extract (10:1) was accurately weighed and dissolved in a methanol–water mixture (50:50, *v*/*v*) to obtain a final concentration of 1 mg/mL. The solution was sonicated for 15 min at room temperature to ensure complete dispersion, then filtered through a 0.45 μm PTFE membrane before analysis. Chromatographic evaluation of *Artemisia capillaris* extract was carried out on a reversed-phase C18 column (250 × 4.6 mm, 5 μm) maintained at 30 °C at a flow rate of 1.0 mL/min. Detection was performed with a diode-array detector, monitoring chlorogenic acid at 325 nm and scoparone at 344 nm, corresponding to their respective absorption maxima. Compound identification was achieved by comparing retention times and UV spectral profiles with those of authenticated reference standards. Quantification was performed using external calibration curves over the concentration range of 2–100 μg/mL, which showed satisfactory linearity (R^2^ > 0.995). The results were expressed as milligrams of compound per gram of dry extract.

### 2.2. Agents Preparation

*Artemisia capillaris* extract (herb water extract; extract ratio 10:1), *Boswellia serrata* extract (water/alcohol extract; extract ratio 10:1; titrated 78.50% in total organic acids, of which 10.29% AKBA, 20.58% AβBA, 5.35% AαBA), *Eruca sativa Mill*. extract (leaf extract titrated 10.41% icariin), and Vitamin D3 (all donated by Vivatis Pharma Italy, Gallarate, Italy) were investigated to identify a strategy capable of influencing mechanisms associated with osteoporosis following simulated intestinal absorption. *Artemisia capillaris* extract (*Artemisia*) was tested at concentrations ranging from 25 µg/mL to 100 µg/mL [34], *Boswellia serrata* extract (*Boswellia*) at concentrations ranging from 50 µg/mL to 200 µg/mL [35], *Eruca sativa* Mill. Extract (*Eruca*) at concentrations ranging from 10 µg/mL to 100 µg/mL [36], while Vitamin D3 was used at a concentration of 100 nM [37,38].

All substances used in the experiment were carefully prepared in phenol red-free Dulbecco’s Modified Eagle Medium (DMEM; Merck Life Sciences, Rome, Italy), which was supplemented with 0.5% fetal bovine serum (FBS), 2 mM L-glutamine, and 1% penicillin-streptomycin to ensure optimal conditions (all chemicals were purchased from Merck Life Sciences, Rome, Italy). Initially, each compound was dissolved at a concentration 10 times the intended final concentration to facilitate accurate dilution and minimise variability. These stock solutions were then meticulously diluted to a series of concentrations to evaluate their biological activity. Each agent was tested alone and in combination (defined as the Mix, corresponding to the Erucalix^®^ formulation) to investigate the potential of a novel multi-component formulation.

In addition, 1 µM dexamethasone was added directly to the culture medium to mimic osteoporosis. This concentration was selected based on previous studies demonstrating reproducible induction of osteoporotic-like cellular alterations without overt cytotoxicity [39].

### 2.3. Cell Culture

Human colorectal adenocarcinoma Caco-2 cells (ATCC, Manassas, VA, USA) were used as an established in vitro model of the intestinal epithelium. Cells were cultured in Advanced Dulbecco’s Modified Eagle Medium (Adv-DMEM; Gibco^®^, Thermo Fisher Scientific, Waltham, MA, USA) supplemented with 10% fetal bovine serum (FBS; Merck Life Science, Rome, Italy), 2 mM L-glutamine, and 1% penicillin–streptomycin (both purchased by Merck Life Science, Rome, Italy). Cultures were maintained at 37 °C in a humidified atmosphere containing 5% CO_2_ [40].

For cell viability assays, 1 × 10^4^ cells were seeded in 96-well plates and analysed using the MTT method. For permeability and barrier integrity studies, 2 × 10^4^ cells were seeded onto 6.5 mm Transwell^®^ inserts (0.4 μm pore size, polycarbonate membrane; Corning^®^ Costar^®^, Merck Life Science, Rome, Italy) placed in 24-well plates. Cells were allowed to differentiate for 21 days, with medium replacement every other day on both apical and basolateral compartments. Prior to treatment, the apical medium was adjusted to pH 6.5 to simulate intestinal luminal conditions, while the basolateral compartment was maintained at pH 7.4, corresponding to physiological blood pH [41].

The MC3T3-E1 murine preosteoblastic cell line (ATCC, Manassas, VA, USA) was cultured under similar conditions in Adv-DMEM supplemented (Gibco^®^, Thermo Fisher Scientific, Waltham, MA, USA) with 10% FBS, 2 mM L-glutamine, and 1% penicillin–streptomycin (Merck Life Science, Rome, Italy). Cells were maintained at 37 °C and 5% CO_2_, with medium replacement every 2–3 days, and passages up to number 23 were used [42,43]. For experimental procedures, cells were seeded at a density of 1 × 10^4^ cells/well in 96-well plates for MTT analysis, 2 × 10^4^ cells/well in 24-well plates for alkaline phosphatase (ALP) activity assays (Merck Life Science, Rome, Italy), and 8 × 10^4^ cells/well in 6-well plates for calcium deposition (Alizarin Red staining; Merck Life Science, Rome, Italy) and intracellular signalling analyses.

RAW264.7 murine macrophages (ATCC, Manassas, VA, USA) were cultured in Adv-DMEM supplemented (Gibco^®^, Thermo Fisher Scientific, Waltham, MA, USA) with 10% FBS, 2 mM L-glutamine, and 1% penicillin–streptomycin (all obtained by Merck Life Science, Rome, Italy) under standard conditions (37 °C, 5% CO_2_) [44]. This cell line is widely used for osteoclast differentiation studies due to its ability to form multinucleated, TRAP-positive osteoclast-like cells upon stimulation [45]. Cells were used at appropriate passage numbers (>18) [46,47] and seeded at a density of 5 × 10^5^ cells/cm^2^ for differentiation experiments [48]. Osteoclastogenesis was induced in medium supplemented with 10 ng/mL RANKL and 10 ng/mL M-CSF (both purchased by Merck Life Science, Rome, Italy), refreshed every 2–3 days [49]. After 5 days, differentiated cells were replated at 4 × 10^4^ cells/well in 24-well plates for TRAP staining (Merck Life Science, Rome, Italy) and signalling analyses, or at 2.5 × 10^5^ cells/well in bone resorption plates to assess resorptive activity.

In all experimental conditions, osteoblasts and osteoclasts were treated with the Caco-2 basolateral fraction (CBF) following pretreatment with 1 µM dexamethasone (Merck Life Science, Rome, Italy).

### 2.4. Experimental Protocol

The study was therefore designed to evaluate the beneficial effects of vitamin D3, the individual plant extracts, and their Mix on human bone cells following simulated oral administration. The investigation was structured into multiple complementary phases aimed at characterising safety, intestinal absorption, and activity on osteogenesis and bone resorption processes. However, prior to undertaking the biological assessments, the extracts of *Artemisia capillaris*, *Eruca sativa* Mill., and *Boswellia serrata* were subjected to detailed phytochemical profiling to identify the main bioactive compounds and establish a solid basis for the subsequent interpretation of the observed biological effects.

Regarding the biological analyses, the study was divided into multiple phases: an intestinal phase, a mature osteoclast phase, and an intestine-osteoblast–preosteoclast axis phase.

Intestinal Phase. To exclude potential cytotoxicity and assess intestinal passage, both single compounds and their combination were evaluated using a Caco-2 cell-based in vitro permeability model. In this preliminary phase, cell viability was assessed using the MTT assay, enabling selection of optimal concentrations without adverse effects. Subsequently, the same formulations were further tested on the in vitro intestinal model to comprehensively evaluate barrier safety and integrity. Specifically, cell viability, intestinal barrier integrity (assessed by transepithelial electrical resistance (TEER)), and tight junction (TJ) analysis via ELISA, and compound absorption (estimated using a fluorescent probe and Jmax calculation) were measured. Basolateral culture media were collected at the end of these stimulations and subsequently used in the following experimental phases.

Mature Osteoclast Phase. To simulate osteoporotic conditions, pre-osteoclastic RAW264.7 cells differentiated into osteoclasts with M-CSF and RANKL were pretreated with 1 µM dexamethasone for three days to induce hyperactivity. This approach enabled subsequent evaluation of TRAP activity, RANK expression, NFκB production, p-p65 expression, and the formation of resorption pits following treatment with the Caco-2 basolateral fraction.

Intestinal–Osteoblast–Preosteoclast Axis Phase. The effects of Caco-2 basolateral fraction were directly tested on MC3T3-E1 osteoblastic cells under osteoporotic conditions induced by treatment with 1 µM dexamethasone for three days [39]. In this phase, cell viability, the expression of osteogenic markers including BMP-2, Runt-Related Transcription Factor 2 (Runx2), RANKL, and OPG, ALP activity, and mineralised nodule formation were assessed. Subsequently, osteoblast-derived digestates were used to treat pre-osteoclasts to investigate indirect effects on osteoclast differentiation. Under these conditions, TRAP activity and RANK and GSK3β expression were measured, providing insights into the potential antiresorptive effects mediated by osteoblasts.

Before treatment, cells were incubated for 8 h in phenol red-free DMEM lacking FBS (Merck Life Science, Rome, Italy) and supplemented with 1% penicillin–streptomycin, 2 mM L-glutamine, and 1 mM sodium pyruvate to synchronise cellular activity.

### 2.5. Intestinal In Vitro Model

A Transwell^®^-based experimental system was employed to reproduce an in vitro intestinal barrier model [50,51,52]. The model relied on differentiated Caco-2 cells, a well-established system widely used to predict intestinal permeability following oral exposure [53,54]. Cells were seeded onto Transwell^®^ inserts (Corning^®^ Costar^®^, Merck Life Science, Rome, Italy) and maintained in complete culture medium for 21–28 days to allow full differentiation. During this period, cells progressively acquired a polarised epithelial phenotype characterised by the formation of tight junctions and apical microvilli.

Barrier integrity was monitored by measuring transepithelial electrical resistance (TEER) every other day using an EVOM3 voltmeter equipped with STX2 electrodes (World Precision Instruments, Sarasota, FL, USA). Experimental treatments were initiated only after TEER values reached ≥400 Ω·cm^2^, indicating the formation of a functionally competent epithelial barrier [55].

Prior to treatment, the apical and basolateral compartments were adjusted to different pH levels to better mimic physiological conditions. Specifically, the apical side was set to approximately pH 6.5, resembling the small intestinal lumen, while the basolateral side was maintained at pH 7.4, corresponding to blood conditions, using 1 M HCl or 1 M NaOH [41]. Cells were then equilibrated for 15 min at 37 °C under 5% CO_2_, and TEER values were re-assessed to confirm barrier stability.

For permeability evaluation, treatments were co-administered with 0.04% fluorescein (Merck Life Science, Rome, Italy) as a fluorescent tracer [56]. Fluorescence in the basolateral compartment was measured using a spectrophotometer (Infinite 200 Pro-MPlex, Tecan, Männedorf, Switzerland) at excitation/emission wavelengths of 490/514 nm. Data were expressed as the percentage of the initial amount applied to the apical side. The permeation rate (J, nmol·min^−1^·mg protein^−1^) was calculated according to previously validated methods using the following equation [56]:

J = (J_max_ × [C])/(K_t_ + [C])

where

C = initial fluorescein concentration.

J_max_ = maximum permeation rate.

K_t_ = Michaelis–Menten constant.

The results are then expressed as mean ± SD (%) compared to the control sample (untreated cells).

Negative controls without cells were analysed to exclude the influence of Transwell membranes.

### 2.6. Cell Viability

Cell viability after treatment was evaluated by MTT assay (Merck Life Science, Rome, Italy), following previously described procedures [57]. Cells were incubated for 2 h at 37 °C in phenol red-free, serum-free DMEM containing 1% MTT reagent. Following incubation, absorbance was measured at 570 nm with background correction at 690 nm using a spectrophotometer (Infinite 200 Pro-MPlex, Tecan, Männedorf, Switzerland).

Data are presented as mean ± SD and expressed as a percentage relative to untreated control cells. Results derive from five independent experiments, each performed in technical triplicate.

### 2.7. Reactive Oxygen Species (ROS) Detection

Intracellular reactive oxygen species (ROS) production was evaluated after treatment by quantifying the release of superoxide anions, as previously described [57]. Absorbance was measured at 550 nm using a spectrophotometer (Infinite 200 Pro-MPlex, Tecan, Männedorf, Switzerland). Results are expressed as mean ± SD and reported as a percentage relative to untreated control cells. Data were obtained from five independent experiments, each performed in technical triplicate.

### 2.8. Claudin-1 Levels Determination

Claudin-1 levels in Caco-2 cell lysates were measured using a commercially available ELISA kit (Cusabio Technology LLC, Houston, TX, USA) following the manufacturer’s instructions [58]. Briefly, cells were lysed in ice-cold PBS and centrifuged at 1500× *g* for 10 min at 4 °C to collect the supernatant. Samples were then processed according to the ELISA protocol, and absorbance was measured at 450 nm using a spectrophotometer (Infinite 200 Pro MPlex, Tecan, Männedorf, Switzerland). Results are expressed as mean ± SD and reported as a percentage relative to untreated control cells. Absolute concentrations were calculated from the standard curve (0–1000 pg/mL) and expressed as pg/mL. Data derived from five independent experiments, each performed in technical triplicate.

### 2.9. Occludin Levels Determination

Occludin content in Caco-2 cell lysates was measured using a Human Occludin ELISA kit (MyBiosource, San Diego, CA, USA) according to the manufacturer’s protocol [58]. Cells were lysed in PBS, and the resulting samples were processed following the assay instructions. Absorbance was recorded at 450 nm using a spectrophotometer (Infinite 200 Pro MPlex, Tecan, Männedorf, Switzerland). Quantitative values were obtained from a standard curve (0–1500 pg/mL) and expressed as pg/mL. Data are presented as mean ± SD and reported as percentage relative to untreated control cells. Results derive from five independent experiments, each performed in technical triplicate.

### 2.10. Zonula Occluden-1 (ZO-1) Levels Determination

ZO-1 levels in Caco-2 cell lysates were quantified using a Human Tight Junction Protein 1 (ZO-1) ELISA kit (MyBiosource, San Diego, CA, USA) following the manufacturer’s protocol [58]. Cells were lysed by repeated freeze–thaw cycles, then washed with cold PBS and centrifuged at 5000× *g* for 5 min at 4 °C to collect the supernatant. Samples were processed according to the ELISA instructions, and absorbance was measured at 450 nm using a spectrophotometer (Infinite 200 Pro MPlex, Tecan, Männedorf, Switzerland). Concentrations were calculated from a standard curve (0–1000 pg/mL) and expressed as pg/mL. Data are presented as mean ± SD and reported as a percentage relative to untreated control cells. Results are derived from five independent experiments, each performed in technical triplicate.

### 2.11. ALP Activity Assay

Alkaline phosphatase (ALP) activity was assessed after 14 days of treatment to evaluate osteoblast function, following previously described procedures [43]. Cells were washed with cold PBS and lysed in buffer containing 50 mM Tris-HCl (pH 7.4) and 1% Triton X-100. Lysates were centrifuged at 14,000 rpm for 20 min at 4 °C, and the supernatants were collected for subsequent analysis. ALP activity was determined using 4-nitrophenyl phosphate (4NPP; 4 mg/mL) as substrate, prepared in 0.2 M 2-amino-2-methyl-1-propanol containing 4 mM MgCl_2_, and incubated for 30 min at 37 °C (all reagents were obtained from Merck Life Science, Rome, Italy). The reaction was terminated by adding 0.1 M NaOH, and absorbance was measured at 405 nm using a spectrophotometer (Infinite 200 Pro MPlex, Tecan, Männedorf, Switzerland). Protein concentration was quantified in parallel using the Pierce BCA Protein Assay Kit (Thermo Fisher Scientific, Waltham, MA, USA). ALP activity values were normalised to total protein content and expressed as a percentage relative to untreated control cells.

### 2.12. Alizarin Red Staining and Quantitative Analysis of Mineralisation

After 21 days of treatment, matrix mineralisation in MC3T3-E1 cells was evaluated by Alizarin Red staining, following previously described procedures [59]. Cells were washed with PBS and fixed in 75% ethanol for 30 min at 4 °C. After fixation, samples were rinsed with distilled water and incubated with 1% Alizarin Red solution (pH 4.2) for 30 min at 37 °C. Excess dye was removed by repeated washing with distilled water, and mineralised nodules were visualised under a low-magnification microscope and documented using an imaging system (DMi1, Leica, Wetzlar, Germany). For quantitative analysis, 1 mL of 100 mM cetylpyridinium chloride was added to each well, and the mixture was incubated for 1 h to solubilise the calcium-bound dye (all reagents were obtained from Merck Life Science, Rome, Italy). The absorbance of the extracted dye was measured at 570 nm using a spectrophotometer (Infinite 200 Pro MPlex, Tecan). Results are expressed as a percentage relative to untreated control cells. 

### 2.13. TRAP Staining and the TRAP Activity Assay

Osteoclast activity was evaluated by tartrate-resistant acid phosphatase (TRAP) staining with a commercial kit (Merck Life Science, Rome, Italy), following the manufacturer’s instructions [60]. RAW264.7-derived osteoclasts were fixed with a citrate buffer/acetone solution (1:1) and then stained with the TRAP reagents. Cells with three or more nuclei were identified as TRAP-positive, and the percentage of multinucleated cells was quantified for each condition. Representative images were acquired with a microscope (DMi1, Leica, Wetzlar, Germany) to document the effects of the treatments. TRAP enzymatic activity was further assessed using para-nitrophenyl phosphate (pNPP) as substrate (Merck Life Science, Rome, Italy). The yellow-coloured product was measured at 405 nm using a spectrophotometer (Infinite 200 Pro MPlex, Tecan, Männedorf, Switzerland). Results are expressed as a percentage relative to untreated control cells.

### 2.14. Biomimetic Calcium Phosphate Assay and Resorption Pit Assay

Osteoclastic resorptive activity was assessed using bone resorption assay plates and fluorescein-labelled Ca^2+^-coated 24-well plates (PG Research, Tokyo, Japan) according to the manufacturer’s instructions [61]. At the end of the treatment period, 100 μL of cell culture supernatant was collected and transferred to a 96-well plate. Subsequently, 50 μL of bone resorption assay buffer was added to each well. Fluorescence intensity was measured using a microplate reader (Infinite 200 Pro MPlex, Tecan, Männedorf, Switzerland) at excitation and emission wavelengths of 485 and 535 nm, respectively. Results are expressed as a percentage relative to untreated control cells.

### 2.15. OPG ELISA Kit

Osteoprotegerin (OPG) levels were quantified using the OPG/TNFRSF11B DuoSet ELISA kit (R&D Systems, Minneapolis, MN, USA) according to the manufacturer’s instructions [62]. Absorbance was measured at 450 nm with a spectrophotometer (Infinite 200 Pro MPlex, Tecan, Männedorf, Switzerland). Concentrations were interpolated from a standard curve spanning 62.5 to 4000 pg/mL. Data are expressed as a percentage relative to untreated control cells.

### 2.16. RANKL ELISA Kit

Receptor activator of nuclear factor kappa-B ligand (RANKL) levels were measured using a Mouse RANKL ELISA kit (Abcam, Cambridge, UK) following the manufacturer’s instructions [63]. Absorbance was measured at 450 nm with a spectrophotometer (Infinite 200 Pro MPlex, Tecan, Männedorf, Switzerland). Concentrations were interpolated from a standard curve spanning 2.74 to 2000 pg/mL. Data are presented as mean ± SD and reported as a percentage relative to untreated control cells.

### 2.17. NF-κB ELISA Kit

NF-κB transcriptional activity was assessed by measuring its DNA-binding capacity using a commercial ELISA kit (Cayman Chemical Company, Ann Arbour, MI, USA), following the manufacturer’s guidelines. Nuclear extracts were obtained through a standard extraction method [56]. NF-κB present in the nuclear fraction was then recognised by a specific primary antibody. The detection stage of the process was performed using an HRP-conjugated secondary antibody, resulting in a colourimetric signal. The signal was measured at 450 nm using a microplate reader (Infinite 200 Pro MPlex, Tecan, Männedorf, Switzerland). NF-κB levels were calculated from the corresponding standard curve and reported as mean ± SD, expressed as a percentage relative to untreated control cells.

### 2.18. Western Blot

At the end of each treatment, both cell types were rinsed with ice-cold PBS supplemented with 2 mM sodium orthovanadate and lysed in ice-cold Complete Tablet Buffer (Roche, Milan, Italy) containing 2 mM sodium orthovanadate, 1 mM phenylmethanesulfonyl fluoride (PMSF; Merck Life Science, Rome, Italy), phosphatase inhibitor cocktail (1:50; Merck Life Science, Rome, Italy), and protease inhibitor cocktail (1:200; Calbiochem, San Diego, CA, USA). Cell lysates were clarified by centrifugation at 14,000× *g* for 20 min at 4 °C. Equal amounts of protein (30 μg) were separated by 10% SDS-PAGE and transferred to polyvinylidene difluoride (PVDF) membranes (Thermo Fisher Scientific, Waltham, MA, USA). Membranes were incubated overnight at 4 °C with primary antibodies against BMP2 (1:500), RUNX2 (1:500), RANK (1:500), GSK3β (1:1000), and RELA/NF-κB p65 (1:500) (all antibodies were purchased from Santa Cruz Biotechnology Inc., Dallas, TX, USA). Protein expression levels were normalised to β-actin (1:5000; Merck Life Science, Rome, Italy) as a loading control. Results are presented as mean ± SD and expressed as a percentage relative to untreated control cells, based on five independent experiments.

### 2.19. Statistical Analysis

All data derive from five independent biological experiments (n = 5), each performed in technical triplicate. Statistical analyses were conducted using GraphPad Prism (version 5). Results are presented as mean ± standard deviation (SD). Group differences were assessed by one-way analysis of variance (ANOVA) with Bonferroni post hoc testing. Statistical significance was set at *p* < 0.05. Interactions among components were further explored using the Bliss independence model, which estimates the expected combined effect under the assumption of independent action of individual compounds. This approach is commonly used to evaluate multicomponent systems.

## 3. Results

### 3.1. Natural Extract Characteristics

Prior to conducting biological experiments, the individual botanical extracts were chemically characterised to evaluate their quality, degree of standardisation, and active constituent content. In relation to the aqueous extract of *Artemisia capillaris*, obtained from the aerial parts at an extraction ratio of 10:1, analysis yielded a reproducible and consistent composition, thereby confirming a tenfold enrichment of the characteristic water-soluble compounds in comparison with the initial plant material. With regard to the hydroalcoholic extract of *Boswellia serrata* (extraction ratio 10:1), a high total organic acid content was observed (78.50%), in addition to a marked presence of the main acetylated boswellic acids, specifically 10.29% AKBA, 20.58% acetyl-β-boswellic acid, and 5.35% acetyl-α-boswellic acid, indicating a high level of standardisation of the bioactive fraction. Finally, for the leaf extract of *Eruca sativa* Mill., an icariin content of 10.41% was determined, thus confirming the efficacy of the extraction process in concentrating phenolic compounds of biological interest. In conclusion, the chemical characterisation confirmed the quality and compositional consistency of the extracts (all data were reported in Table 1). This finding supports the hypothesis that the extracts are suitable for subsequent biological evaluations.

### 3.2. Screening Analysis of Individual Natural Extracts and Evaluation of the Effects of the Combination on the Intestine

Following characterisation of the chemical composition of the individual extracts, dose–response analyses were conducted at the intestinal level to identify suitable concentrations for combination with vitamin D_3_ (100 nM).

**Figure 1:**
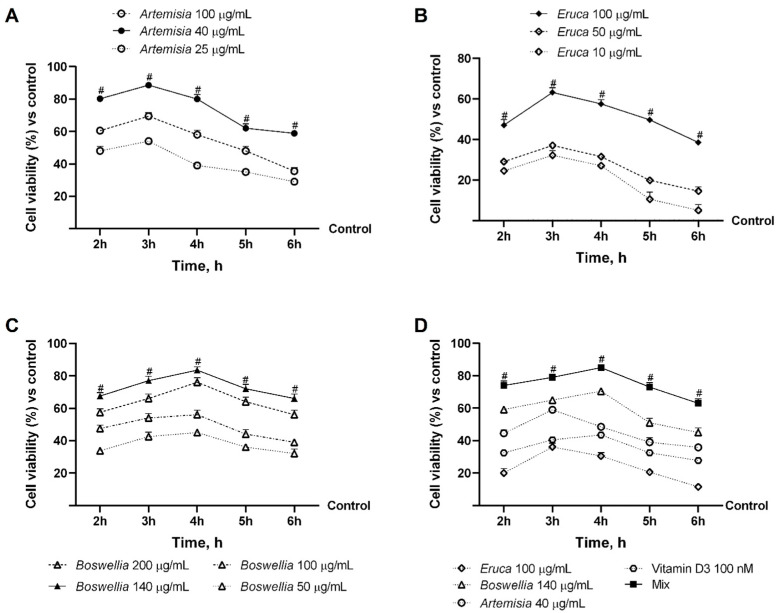
Screening analysis of individual natural extracts and evaluation of the effects of the combination on the intestine. In (**A**), dose–response study of *Artemisia* from 2 h to 6 h (range of 25 and 100 μg/mL); in (**B**), dose–response study of *Eruca* from 2 h to 6 h (range of 10 and 100 μg/mL); in (**C**), dose–response study of *Boswellia* from 2 h to 6 h (range of 50 and 200 μg/mL); and in (**D**), cell viability analysis performed by MTT test. Results are expressed as mean ± SD (%) of 5 biological replicates (n = 5) normalised to the control (untreated sample, 0%). Statistical analysis was performed using one-way ANOVA followed by Bonferroni’s post hoc test for multiple comparisons. Mix = *Artemisia* 40 μg/mL + *Eruca* 100 μg/mL + *Boswellia* 140 μg/mL + Vitamin D3 100 nM. In (**A**–**C**), *p* < 0.05 vs. control; # *p* < 0.05 vs. other concentrations. In (**D**), *p* < 0.05 vs. control; # *p* < 0.05 vs. single agents.

As shown in Figure 1A, *Artemisia capillaris*, tested at 25–100 μg/mL, significantly increased cell viability compared with control conditions (*p* < 0.05), with no evidence of cytotoxicity. Among the tested concentrations, 40 μg/mL showed the most consistent effect over time, with a peak response at 3 h, yielding higher viability than at 25 μg/mL or 100 μg/mL (*p* < 0.05).

Similarly, *Eruca sativa* (Figure 1B) exhibited a concentration-dependent increase in cell viability, with all tested doses significantly higher than control (*p* < 0.05). The strongest effect was observed at 100 μg/mL, particularly at 3 h, with a more pronounced increase than at lower concentrations (*p* < 0.05).

For *Boswellia serrata* (Figure 1C), all tested concentrations (50–200 μg/mL) significantly enhanced cell viability compared with the control (*p* < 0.05), with a maximal effect observed at 4 h. Among these, 140 μg/mL provided the most consistent response, showing higher activity than at both lower and higher concentrations (*p* < 0.05).

**Figure A1:**
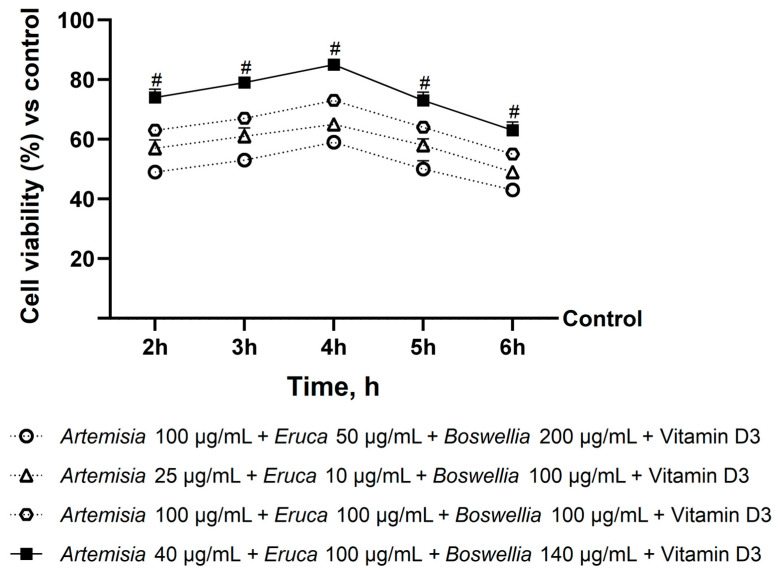
Screening analysis of a combination of natural extracts. Cell viability analysis performed by MTT test. Results are expressed as mean ± SD (%) of 5 biological replicates (n = 5) normalised to the control (untreated sample, 0%). Statistical analysis was performed using one-way ANOVA followed by Bonferroni’s post hoc test for multiple comparisons. *p* < 0.05 vs. control; # *p* < 0.05 vs. other combinations.

Preliminary screening of different combinations (Appendix A, Figure A1) revealed a time-dependent effect on cell viability, with a peak response at 4 h followed by a gradual decline. Based on these data, the combination of *Artemisia* (40 μg/mL), *Eruca* (100 μg/mL), *Boswellia* (140 μg/mL), and vitamin D_3_ (100 nM) was selected for further investigation, as it showed the most consistent effect across time points compared with alternative ratios.

As reported in Figure 1D, all individual components at the selected concentrations significantly increased cell viability compared with the control (*p* < 0.05). When combined, the Mix produced a greater overall effect than any individual component, particularly at 4 h, suggesting a combined, potentially additive, contribution of the components under the tested conditions.

### 3.3. Assessment of the Safety and Absorption of Individual Agents and Their Combination in an In Vitro Intestinal Model

**Figure 2:**
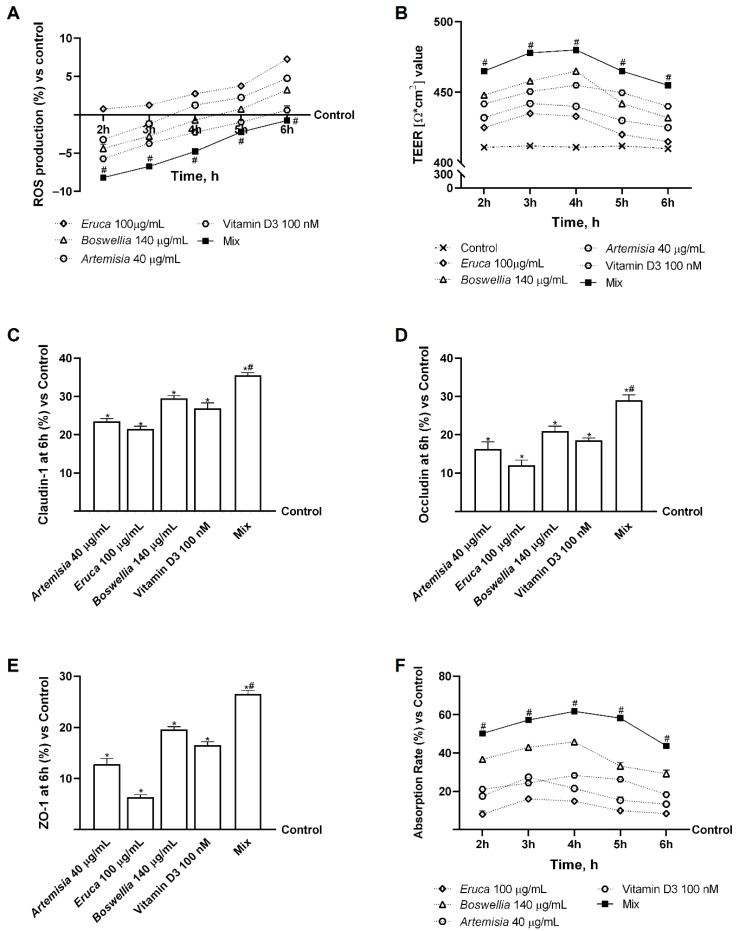
Assessment of the safety and absorption of individual agents and their combination in an in vitro intestinal model. In (**A**), ROS production from 2 h to 6 h was measured by Cytochrome C reduction; in (**B**), TEER values measurement by EVOM3; in (**C**), Claudin-1 levels evaluated by ELISA kit; in (**D**), Occludin levels evaluated by ELISA kit; in (**E**), Zo-1 levels evaluated by ELISA kit; and in (**F**), absorption analysis was evaluated by fluorescein 0.04% probe. Results are expressed as mean ± SD (%) of 5 biological replicates (n = 5) normalised to the control (untreated sample, 0%). Statistical analysis was performed using one-way ANOVA followed by Bonferroni’s post hoc test for multiple comparisons. Mix = *Artemisia* 40 μg/mL + *Eruca* 100 μg/mL + *Boswellia* 140 μg/mL + Vitamin D3 100 nM. In (**A**,**B**,**F**), *p* < 0.05 vs. control; # *p* < 0.05 vs. single agents. In (**C**–**E**), * *p* < 0.05 vs. control; # *p* < 0.05 vs. single agents.

In an in vitro model of the intestinal barrier, further studies were conducted to evaluate the effects of individual agents, both in isolation and in combination. To verify the safety of these molecules, the ROS production, TEER values, TJ levels, and intestinal absorption of the individual agents and their combination (Jmax) were examined. This was done to demonstrate the individual agents’ ability to work cooperatively. As illustrated in Figure 2A, the results pertaining to ROS production following treatment with individual agents and their combination revealed that the individual agents were capable of sustaining ROS levels within physiological parameters (within 10% of the control value), while the combination demonstrated the capacity to maintain ROS levels below control values between 2 h and 4 h (*p* < 0.05). Subsequent analyses assessed intestinal barrier integrity using TEER values. As shown in Figure 2B, TEER analysis revealed that all substances examined maintained adequate intestinal homeostasis (*p* < 0.05). Specifically, integrity analyses demonstrated that 40 μg/mL of *Artemisia*, 100 μg/mL of *Eruca*, 140 μg/mL of *Boswellia*, and 100 nM of Vitamin D3 alone could maintain epithelial integrity by increasing paracellular ion exchange flow through the intestinal epithelium to a greater extent than the control (*p* < 0.05). At the same time, Mix was found to enhance the efficacy of the individual agents (*p* < 0.05). The veracity of these data was confirmed by analysis of TJ levels (see Figure 2C–E). The individual agents alone exerted the most significant effects relative to the control group for all TJs tested (*p* < 0.05). Furthermore, Mix demonstrated the most significant effect in comparison to the individual agents with regard to claudin-1 (approximately 34% compared to *Artemisia*, 39% compared to *Eruca*, 17% compared to *Boswellia* and 24% compared to Vitamin D3; *p* < 0.05), occludin (approximately 44% compared to *Artemisia*, 59% compared to *Eruca*, 28% compared to *Boswellia* and 36% compared to Vitamin D3; *p* < 0.05) and Zo-1 (approximately 52% compared to *Artemisia*, 76% compared to *Eruca*, 26% compared to *Boswellia* and 38% compared to Vitamin D3; *p* < 0.05).

Subsequent experiments were conducted to evaluate the absorption rate of the individual agents and Mix through the intestinal barrier to the final target, using a fluorescent probe. The data obtained using basolateral environment analysis, as illustrated in Figure 2F, support the results previously obtained, as the individual agents demonstrated greater absorption in comparison to the control (*p* < 0.05) with a peak absorption at 3 h for *Artemisia* and *Eruca* and 4 h for *Boswellia* and Vitamin D3 (*p* < 0.05). Furthermore, Mix demonstrated maximum absorption after 4 h of treatment, exhibiting a significant percentage increase of approximately 65% compared to *Artemisia* (*p* < 0.05), 76% compared to *Eruca* (*p* < 0.05), 26% compared to *Boswellia* (*p* < 0.05) and 54% compared to Vitamin D3 (*p* < 0.05). In addition, the chemical composition of the fraction collected from the basolateral compartment was analysed to identify compounds that are effectively available after intestinal passage. The results indicate that this fraction does not simply reflect the original composition of the crude extracts but includes a range of secondary metabolites generated through cellular biotransformation. As reported in Table A1 (Appendix A), measurable levels of bioactive isothiocyanates (approximately 1.4 μg/mL), flavonoid aglycones (~0.9 μg/mL), and modified terpenoid derivatives (approximately 1.3 μg/mL) were detected, together with free vitamin D_3_ (approximately 1.1 nM). These findings suggest that the intestinal barrier contributes to the transformation of initial phytocompounds, generating a pool of bioavailable molecules that may modulate downstream cellular pathways.

### 3.4. Effect of Single Agents and Mix on Osteoclasts in Dexamethasone-Induced Osteoporosis

To determine whether all tested substances can counteract the excessive osteoclast activity characteristic of osteoporotic conditions, the Caco-2 basolateral fraction was used to treat osteoclasts (RAW264.7 monocytic cells differentiated with RANKL and M-CSF). Osteoclast activity was assessed by evaluating TRAP activity, RANK expression, the inflammatory profile (NF-κB and p-p65), and bone resorption activity (bone pit assay).

**Figure 3:**
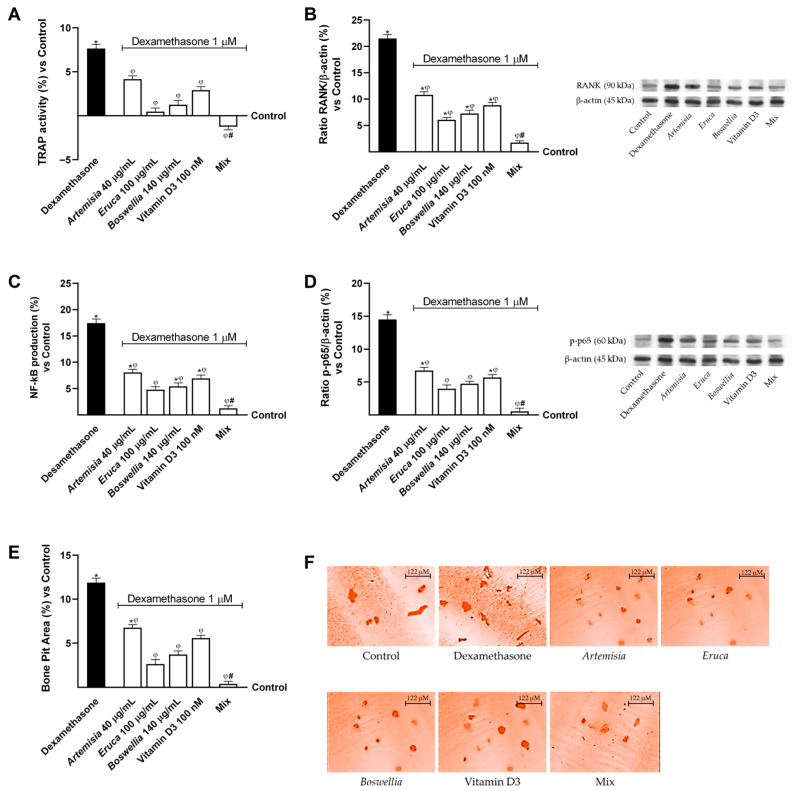
Effect of individual agents and Mix on osteoclasts in dexamethasone-induced osteoporosis. In (**A**), TRAP activity was evaluated by specific assay kit; in (**B**), RANK expression measured by Western blot analysis; in (**C**), NFkB production was quantified by ELISA Kit; in (**D**), p-p65 expression measured by Western blot analysis; in (**E**), Bone resorption pit area quantified using a biomimetic calcium phosphate assay; and in (**F**), an example of staining of bone resorption pit area at 10x magnification (122 μM). Data were expressed as mean ± SD (%) of 5 biological replicates (n = 5; performed in triplicate), normalized to the control (untreated sample, 0%). Statistical analysis was performed using one-way ANOVA followed by Bonferroni’s post hoc test for multiple comparisons. Dexamethasone = osteoporotic condition; Mix = *Artemisia* 40 μg/mL + *Eruca* 100 μg/mL + *Boswellia* 140 μg/mL + Vitamin D3 100 nM. * *p* < 0.05 vs. control; φ *p* < 0.05 vs. Dexamethasone; # *p* < 0.05 vs. single agents.

First, osteoclast precursor cells were differentiated with RANKL and M-CSF and subsequently treated with dexamethasone to enhance osteoclastic activity [64]. As shown in Figure 3A, TRAP activity was significantly increased in osteoclasts following dexamethasone treatment compared with the control (about 7.65% vs. control, *p* < 0.05). Conversely, treatment with the Caco-2 basolateral fraction significantly reduced dexamethasone-induced osteoclastic hyperactivity (about 45% for *Artemisia*, *p* < 0.05; about 94% for *Eruca*, *p* < 0.05; about 84% for *Boswellia*, *p* < 0.05; and about 62% for vitamin D3, *p* < 0.05). Moreover, the Mix exerted a stronger effect than the individual compounds, as it reduced TRAP activity by approximately 4.3-fold compared with *Artemisia* (*p* < 0.05), 1.38-fold compared with *Eruca* (*p* < 0.05), 2-fold compared with *Boswellia* (*p* < 0.05), and 3.3-fold compared with Vitamin D3 (*p* < 0.05). In addition, these results suggest a potential synergistic effect among the tested substances. To quantitatively assess the interaction among the components, Bliss independence analysis was performed. Synergy was calculated using the Bliss independence equation, and the corresponding values are reported in Table A2 in Appendix A.

Similarly, RANK levels on the surface of osteoclasts were significantly increased in osteoporotic conditions compared with controls (about 21.5% vs. controls, *p* < 0.05; Figure 3B). Conversely, the individual agents, following intestinal metabolism, reduced RANK expression on osteoclasts, with a more pronounced effect observed when the compounds were combined. Specifically, the Mix reduced RANK expression by approximately 84% compared with *Artemisia* (*p* < 0.05), 71% compared with *Eruca* (*p* < 0.05), 76% compared with *Boswellia* (*p* < 0.05), and 80% compared with vitamin D3 (*p* < 0.05).

Consistent with the increased expression of RANK in osteoclasts, an enhancement of inflammatory signalling was also observed, as assessed by the levels of NF-κB and p-p65 (Figure 3C,D). In this case as well, the individual agents were able to counteract dexamethasone-induced effects (*p* < 0.05). However, the most pronounced effect was observed following treatment with the Mix, which resulted in reductions of approximately 84.5% and 92% compared with *Artemisia* (*p* < 0.05), 74% and 86% compared with *Eruca* (*p* < 0.05), 77% and 88% compared with *Boswellia* (*p* < 0.05), and 82% and 90% compared with vitamin D3 (*p* < 0.05).

In conclusion, to further confirm the inhibitory effects of the treatments under investigation on osteoclastic activity, the bone resorption capacity of osteoclasts under osteoporotic conditions was evaluated (Figure 3E,F). Based on the data reported in Figure 3E, dexamethasone significantly increased p-p65 expression compared with the control (about 12% vs. control, *p* < 0.05). Conversely, the individual agents reduced the expression of this protein compared with dexamethasone (*p* < 0.05). Notably, the strongest effect was observed following treatment with the Mix, which reduced bone pit resorption by approximately 94% compared with *Artemisia* (*p* < 0.05), 85% compared with *Eruca* (*p* < 0.05), 89% compared with *Boswellia* (*p* < 0.05), and 93% compared with vitamin D3 (*p* < 0.05).

### 3.5. Effect of Mix on Intestinal-Osteoblast-Undifferentiated Osteoclast Axis in Dexamethasone-Induced Osteoporosis

Bone mass reduction is associated with a physiological decline in osteoblastic activity, leading to a decreased rate of bone mineral apposition, followed by an increase in adipocyte and osteoclast numbers, and enhanced bone resorption. Therefore, to investigate the impact of the tested natural extracts on osteoporotic alterations in osteoblastic homeostasis, their effects were evaluated by analysing differentiation and maturation processes through assessment of cell viability, BMP2 and Runx2 expression, and RANKL and OPG levels.

For this purpose, these parameters were assessed after treating osteoblastic cells for 21 days with Caco-2 basolateral fraction, following the induction of osteoporotic conditions by pre-treatment with dexamethasone for three days. Accordingly, this phase of the study evaluated the effects of the tested substances on osteoblastic activity under osteoporotic conditions and on monocyte differentiation into osteoclasts.

**Figure 4:**
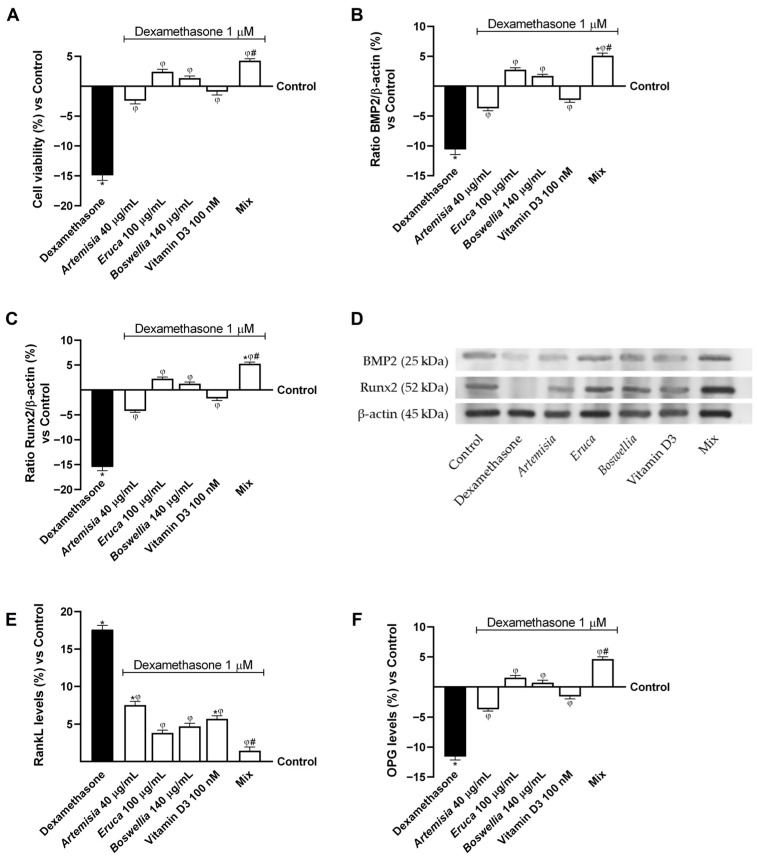
Effect of Mix on the intestinal-osteoblast axis in dexamethasone-induced osteoporosis. In (**A**), cell viability was measured by MTT test; in (**B**), BMP2 densitometric analysis of the specific Western blot; in (**C**), Runx2 densitometric analysis of the specific Western blot; in (**D**), an example of Western blot lane of each parameters were reported; in (**E**), RANKL levels quantification allowed by specific ELISA Kit; and in (**F**), OPG levels evaluated by ELISA Kit. Results were expressed as mean ± SD (%) of 5 biological replicates (n = 5), each performed in triplicate, normalised to the control (untreated sample, 0%). Statistical analysis was performed using one-way ANOVA followed by Bonferroni’s post hoc test for multiple comparisons. Dexamethasone = osteoporotic condition; Mix = *Artemisia* 40 μg/mL + *Eruca* 100 μg/mL + *Boswellia* 140 μg/mL + Vitamin D3 100 nM. * *p* < 0.05 vs. control; φ *p* < 0.05 vs. Dexamethasone; # *p* < 0.05 vs. single agents.

First, osteoblast viability under dexamethasone-induced osteoporotic conditions (1 μM) was evaluated (Figure 4A). The data revealed that a three-day treatment with dexamethasone significantly reduced cell viability by approximately 15% compared with the control (*p* < 0.05). Conversely, following intestinal metabolism, the individual agents were able to reverse the negative effects induced by dexamethasone, restoring osteoblastic cell viability by approximately 84% (*Artemisia* vs. dexamethasone, *p* < 0.05), 1.16-fold (*Eruca* vs. dexamethasone, *p* < 0.05), 1.09-fold (*Boswellia* vs. dexamethasone, *p* < 0.05), and 94% (vitamin D3 vs. dexamethasone, *p* < 0.05). In addition, when combined, the effects of the individual agents were amplified, resulting in a significantly greater effect than that observed with each single compound alone, with increases of approximately 1.56-fold compared with *Artemisia* (*p* < 0.05), 43.5% compared with *Eruca* (*p* < 0.05), 68% compared with *Boswellia* (*p* < 0.05), and 1.21-fold compared with vitamin D3 (*p* < 0.05).

Similarly, a comparable trend was observed for BMP2 and Runx2 expression following treatment with all the tested agents (Figure 4B,C). Specifically, dexamethasone treatment significantly reduced BMP2 and Runx2 expression compared with the control (*p* < 0.05); however, this reduction was reversed following treatment with the individual agents (*p* < 0.05). Notably, the Mix exerted a greater beneficial effect than the single compounds, increasing the expression of both proteins by approximately 1.9-fold and 1.8-fold compared with *Artemisia* (*p* < 0.05), 46% and 57% compared with *Eruca* (*p* < 0.05), 67% and 76% compared with *Boswellia* (*p* < 0.05), and 1.45-fold and 1.32-fold compared with vitamin D3 (*p* < 0.05).

Finally, RANKL and OPG levels were evaluated following treatment with the investigated agents to restore the proper balance between these two factors. Indeed, under osteoporotic conditions, an imbalance occurs between RANKL and OPG release, favouring RANKL, which promotes the differentiation of pre-osteoclastic cells into osteoclasts, thereby enhancing bone resorption and leading to bone matrix degradation. Based on the data shown in Figure 4E,F, cells exposed to dexamethasone-induced osteoporotic conditions exhibited a significant increase in RANKL secretion (+18% vs. control, *p* < 0.05) and a concomitant reduction in OPG production (about −12% vs. control, *p* < 0.05) compared with the control. Conversely, all individual agents were able to reverse the negative effects induced by dexamethasone by reducing RANKL levels by approximately 57% for *Artemisia* (*p* < 0.05), 78% for *Eruca* (*p* < 0.05), 73% for *Boswellia* (*p* < 0.05), and 68% for vitamin D3 (*p* < 0.05). Regarding OPG levels, the individual agents significantly increased OPG production compared with dexamethasone by approximately 68% (*Artemisia* vs. dexamethasone, *p* < 0.05), 1.1-fold (*Eruca* vs. dexamethasone, *p* < 0.05), 1.06-fold (*Boswellia* vs. dexamethasone, *p* < 0.05), and 86.5% (vitamin D3 vs. dexamethasone, *p* < 0.05).

For both parameters, the most pronounced effect against osteoporotic conditions was observed following treatment with the Mix, which exceeded the effects of the individual agents by approximately 81% and 1.8-fold compared with *Artemisia* (*p* < 0.05), 62% and 66.5% compared with *Eruca* (*p* < 0.05), 69% and 85% compared with *Boswellia* (*p* < 0.05), and 74.5% and 1.34-fold compared with vitamin D3 (*p* < 0.05).

**Figure 5:**
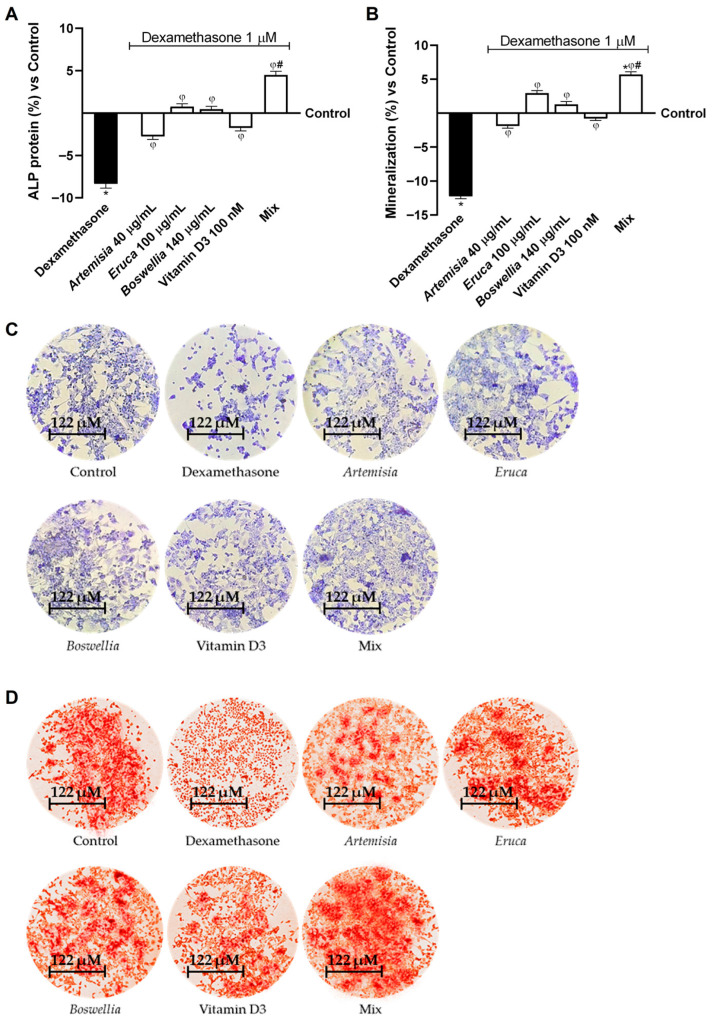
Effect of Mix on osteoblast maturation and mineralization under dexamethasone-induced osteoporotic conditions. In (**A**), ALP protein quantification using a specific kit; in (**B**), mineralisation analysis performed by Alizarin red assay; in (**C**), an example of ALP staining at 10x magnification (122 μM); and in (**D**), an example of Alizarin red staining with calcium deposits at 10x magnification (122 μM). Results are expressed as mean ± SD (%) of 5 biological replicates (n = 5; performed in triplicate) normalised to the control (untreated sample, 0%). Statistical analysis was performed using one-way ANOVA followed by Bonferroni’s post hoc test for multiple comparisons. Dexamethasone = osteoporotic condition; Mix = *Artemisia* 40 μg/mL + *Eruca* 100 μg/mL + *Boswellia* 140 μg/mL + Vitamin D3 100 nM. * *p* < 0.05 vs. control; φ vs. Dexamethasone; # *p* < 0.05 vs. single agents.

Furthermore, to verify that the investigated agents, including Mix, can improve osteoporotic conditions by enhancing osteoblastic activity, ALP levels and bone mineralisation were evaluated as markers of osteoblast maturation and bone matrix integrity. As shown in Figure 5A (with representative staining in Figure 5C), ALP protein levels were markedly reduced compared with the control (about −8.4% vs. control, *p* < 0.05). However, the individual treatments reversed the dexamethasone-induced impairment, restoring ALP levels to levels comparable to those in the control. Moreover, when combined, the effects of the individual agents were significantly enhanced, as the Mix increased ALP levels by approximately 1.61-fold (83%), 1.4-fold (90%), and 1.4-fold (vitamin D3), respectively, suggesting a synergistic interaction among the components. Accordingly, synergy was confirmed using the Bliss independence equation, and the corresponding values are reported in Table A3 (Appendix A).

Similarly, mineralisation levels were negatively modulated following dexamethasone-induced osteoporotic conditions (about −12.3% vs. control, *p* < 0.05; Figure 5B; specific staining shown in Figure 5D). In addition, in this case, the individual agents were able to counteract this condition, restoring mineralisation levels to those of the control; however, the Mix exerted the strongest effect. Indeed, synergy analysis using the Bliss equation (Table A4 in Appendix A) revealed a greater effect of approximately 1.33-fold compared with *Artemisia* (*p* < 0.05), 48% compared with *Eruca* (*p* < 0.05), 77% compared with *Boswellia* (*p* < 0.05), and 1.14-fold compared with vitamin D3 (*p* < 0.05).

Finally, to further evaluate whether the beneficial effects of the individual agents and the Mix on the osteoporotic process could be mediated through osteoblast–osteoclast crosstalk, conditioned media derived from osteoblastic cells under osteoporotic conditions and treated with the investigated agents were used to treat pre-osteoclastic cells. This approach enabled assessment of osteoclast differentiation and confirmation of the previously observed reduction in RANKL release by osteoblasts. Accordingly, TRAP activity and the expression of GSK3β and the RANK receptor were evaluated in pre-osteoclastic cells.

**Figure 6:**
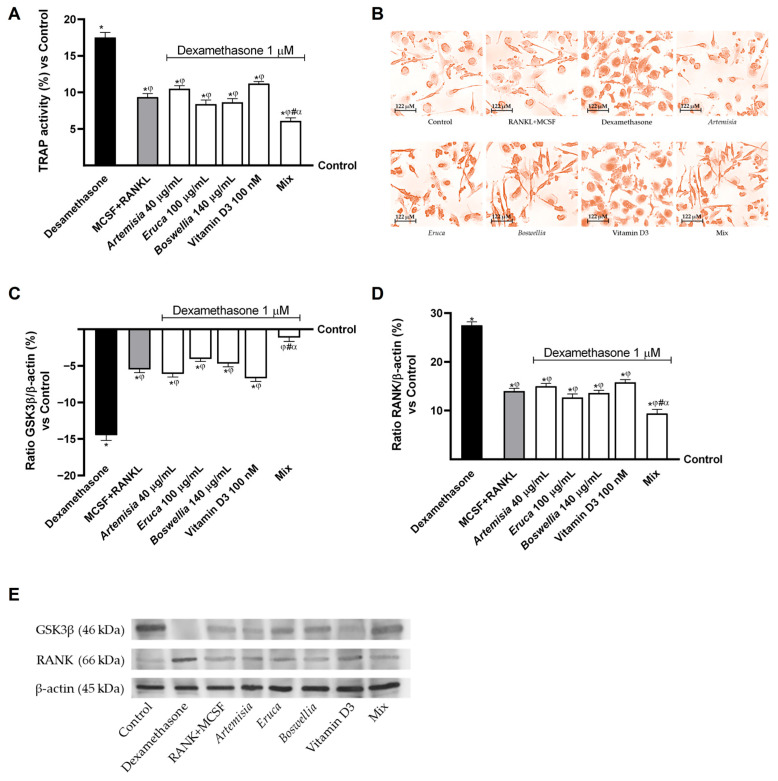
Effect of Mix on intestinal-osteoblast-undifferentiated osteoclast axis in dexamethasone-induced osteoporosis. In (**A**), TRAP activity quantification using a specific kit; in (**B**), an example of TRAP staining at 10x magnification (122 μM); in (**C**), GSK3β densitometric analysis of the specific Western blot; in (**D**), RANK densitometric analysis of the specific Western blot; in (**E**), an example of Western blot lane of each parameters were reported. Results are expressed as mean ± SD (%) of 5 biological replicates (n = 5; performed in triplicate) normalised to the control (untreated sample, 0%). Statistical analysis was performed using one-way ANOVA followed by Bonferroni’s post hoc test for multiple comparisons. Dexamethasone = osteoporotic condition; MCSF = Macrophage colony-stimulating factor (maturation inducer); RANKL = Receptor activator of NF-κB ligand (maturation inducer); Mix = *Artemisia* 40 μg/mL + *Eruca* 100 μg/mL + *Boswellia* 140 μg/mL + Vitamin D3 100 nM. * *p* < 0.05 vs. control; φ vs. 1 μM Dexamethasone; # *p* < 0.05 vs. single agents, α *p* < 0.05 vs. MCSF + RANKL.

As shown in Figure 6A,B, TRAP activity was significantly increased following treatment with osteoblast-conditioned medium containing dexamethasone compared with the control (*p* < 0.05), confirming that this inducer can increase RANKL levels and promote osteoclast differentiation, exceeding even the effect of the positive differentiation inducer M-CSF + RANKL (*p* < 0.05). Conversely, all individual agents significantly reduced osteoclast differentiation compared with dexamethasone (*p* < 0.05), achieving effects comparable to those exerted by the positive inducer. Moreover, the Mix not only reduced TRAP activity compared with both dexamethasone and the positive inducer but also exerted stronger beneficial effects than the individual agents (about 42% vs. *Artemisia*, *p* < 0.05; about 27% vs. *Eruca*, *p* < 0.05; about 29.5% vs. *Boswellia*, *p* < 0.05; about 45.5% vs. vitamin D3, *p* < 0.05).

Finally, the expression of GSK3β and RANK proteins in pre-osteoclastic cells was evaluated following treatment with the investigated agents (Figure 6C,D). The data showed that dexamethasone markedly reduced GSK3β expression and increased RANK expression to a greater extent than the control (about −14.5% and +27.5%, respectively; *p* < 0.05), confirming the alterations previously observed in osteoblasts under osteoporotic conditions. In contrast, the individual agents were able to counteract the deleterious effects of dexamethasone, limiting pre-osteoclast differentiation by increasing GSK3β expression and reducing RANK expression compared with dexamethasone (*p* < 0.05), with effects comparable to those of the positive osteoclast differentiation inducer (M-CSF + RANKL). In addition, the Mix exerted a stronger effect than the individual agents by increasing GSK3β expression by approximately 81% compared with *Artemisia* (*p* < 0.05), 72% compared with *Eruca* (*p* < 0.05), 76% compared with *Boswellia* (*p* < 0.05), and 83% compared with vitamin D3. Moreover, it reduced RANK expression by approximately 37% compared with *Artemisia* (*p* < 0.05), 26% compared with *Eruca* (*p* < 0.05), 31% compared with *Boswellia* (*p* < 0.05), and 40.5% compared with vitamin D3, showing effects that were also significantly greater than those of the positive differentiation inducer (*p* < 0.05).

## 4. Discussion

The fundamental cause of the increasing imbalance between bone production and resorption that characterises osteoporosis is the dysfunction of osteoblasts and elevated osteoclast activity [65]. The functional link between osteoblasts and osteoclasts tightly regulates bone homeostasis under physiological conditions, with the RANKL/OPG axis playing a key role in controlling osteoclastogenesis [66]. However, in cases of osteoporosis, this equilibrium is disrupted, leading to increased osteoclastic activity, decreased osteoblastic differentiation and mineralisation, and a net loss of bone mass [17].

The concept of the gut–bone axis has been increasingly recognised in recent years, reflecting accumulating evidence of a functional link between intestinal physiology and bone homeostasis. The intestinal barrier is a highly organised and dynamic system that integrates structural, immune, and biochemical components to maintain mucosal integrity and regulate immune responses [67]. Continuous epithelial turnover is a key feature of this system, ensuring barrier adaptability and helping regulate transepithelial permeability. This process is tightly regulated by intercellular junctional complexes, particularly tight junctions (TJs), which play a central role in preserving epithelial integrity [68].

However, a low-grade systemic inflammatory state may be triggered by alterations in intestinal homeostasis, thereby dysregulating bone metabolism. In this regard, the RANKL/OPG axis, nutrient bioavailability essential for bone health, and regulation of osteoclastic activity all play significant roles in the pathophysiology of osteoporosis [69].

The objective of this study was to determine whether a combination of *Artemisia*, *Eruca*, and *Boswellia*, supplemented with vitamin D3 (Mix), has the potential to reverse these pathological changes by influencing key cellular and molecular processes involved in bone remodelling. Specifically, this combination was selected following an extensive preliminary screening, which aimed to identify the optimal ratio of components capable of exerting a synergistic protective effect. Indeed, in the present study, the integrity of the intestinal tract was enhanced by the administration of specific natural extracts and vitamin D3, which maintained decreased ROS production without causing deleterious effects on the intestine. The intestinal barrier converts plant compounds into secondary metabolites, making them bioavailable and capable of activating intracellular signalling pathways. Specifically, Mix significantly decreased ROS generation and more effectively restored epithelial barrier function, exerting the most protective impact. This finding suggests that the components interact favourably, thereby enabling the simultaneous regulation of multiple intestinal targets. In addition, these findings could support the growing evidence of intestinal modulation of the osteoporotic process [70,71,72,73,74].

As previously reported [75,76], the osteoporotic conditions induced by dexamethasone in our model led to a marked increase in osteoclastic activity. The marked increase in TRAP activity, RANK surface expression, and NF-κB signalling observed under dexamethasone treatment confirms the strong pro-osteoclastogenic effect of glucocorticoids. The results are consistent with earlier reports showing that glucocorticoid treatment stimulates osteoclastogenesis and enhances bone resorption via NF-κB-mediated signaling mechanisms [77]. The utilisation of dexamethasone as an inductive agent is a well-established methodological strategy in experimental bone biology. This approach facilitates the precise isolation of the molecular mechanisms that are responsible for cellular damage. This glucocorticoid has been shown to reliably recapitulate the key pathological features associated with glucocorticoid-induced bone loss. These include inhibition of osteoblast proliferation and differentiation, induction of apoptosis in osteoblasts and osteocytes, and stimulation of osteoclast activity [78]. Furthermore, by modulating key signalling pathways, including Wnt/β-catenin, and transcription factors essential for osteogenic maturation, such as RUNX2, dexamethasone induces morphological and functional alterations characteristic of osteopenia and osteoporosis. This experimental approach, when validated, enables investigation of intracellular mechanisms shared across different forms of bone loss. In doing so, it highlights common pathways responsible for bone resorption and dysfunction. Consequently, the data obtained from our model are pertinent not only to understanding the effects of glucocorticoids but also to interpreting the cellular processes underlying more general osteoporotic conditions [79].

On the osteoblastic side, dexamethasone-pretreated cells exhibited a marked suppression of osteoblastic activity, as evidenced by reduced ALP activity, impaired matrix mineralisation, and downregulation of the osteogenic markers BMP2 and Runx2. Under these conditions, individual extracts and vitamin D3 partly mitigated the harmful effects of dexamethasone by enhancing cell viability and proliferation, osteogenic marker expression, ALP activity, and mineralisation. In contrast, the combined formulation, referred to as Mix, displayed a significantly greater effect, enhancing all these parameters and suggesting a synergistic interaction, as confirmed by Bliss independence analysis. The upregulation of BMP2 and Runx2 further supports the role of Mix in promoting osteoblast differentiation through canonical osteogenic pathways, thereby favouring osteoblast commitment and maturation as well as bone matrix mineralisation. These results are consistent with earlier research linking *Boswellia*, *Artemisia*, and *Eruca* to osteoprotective properties [26,35,80].

In the present experimental design, intestinally metabolised fractions obtained from the Caco-2 model were used to more closely simulate the bioavailable pool of nutraceutical compounds that reach bone cellular targets. To assess the ability of the treatments to modulate osteoblast–osteoclast crosstalk, conditioned medium derived from treated osteoblasts was used to stimulate pre-osteoclastic cells. The data showed that conditioned medium containing dexamethasone stimulated TRAP activity and increased RANK expression, whereas treatment with the individual agents and, to a greater extent, with Mix, limited osteoclast differentiation by increasing GSK3β levels and reducing RANK expression compared with dexamethasone. These findings further confirm the beneficial effects of the extracts on the osteoblast–osteoclast microenvironment and are consistent with previously reported evidence on the role of the individual agents in modulating osteoclastogenesis [19,21,81].

From a translational perspective, modulation of the RANKL/OPG ratio represents one of the most relevant findings of this study. Dexamethasone increased RANKL expression and reduced OPG levels in osteoblasts under osteoporotic conditions, thereby disrupting the bone microenvironment and promoting osteoclast activation. These alterations collectively reflect the profound imbalance between bone formation and resorption typically observed in glucocorticoid-induced osteoporosis. In contrast, treatment with the individual extracts and with Mix reversed these effects in both osteoblasts and pre-osteoclasts. In osteoblasts, the treatments increased ALP activity and improved mineralisation, restoring a more osteogenic profile closer to control conditions, as previously reported in the literature [18,26,82]. In contrast, all treatments reduced TRAP activity and RANK expression in pre-osteoclasts while increasing GSK3β levels, thereby limiting osteoclast differentiation. The combined formulation, Mix, exhibited the greatest synergistic efficacy, producing superior effects on both the osteoblastic and pre-osteoclastic compartments. Although effect sizes differed across experimental endpoints, the overall biological direction of the responses consistently supported a synergistic modulation of osteoblast–osteoclast dynamics. These findings are supported by previous evidence indicating that *Boswellia* and *Artemisia* extracts can inhibit TRAP activity and suppress signalling pathways associated with RANK and NF-κB activation [19,21]. Although the magnitude of the observed effects varied across experimental conditions, the overall biological trends consistently indicated a synergistic modulation of osteoblast–osteoclast dynamics. Additionally, the regulation of GSK3β is recognised as a key regulatory node in modulating osteoclast differentiation through signalling pathways such as PI3K/Akt and Wnt/β-catenin, which critically influence the balance between bone formation and bone resorption [83]. In this context, the increase in GSK3β levels observed in pre-osteoclasts following treatment with the individual extracts and Mix suggests a potential contribution of these compounds in limiting osteoclast maturation by negatively modulating pro-osteoclastogenic signalling pathways.

Overall, the results obtained clearly indicate that Mix exerts a marked osteoprotective effect by enhancing osteoblast activity and differentiation, limiting osteoclastogenesis, and restoring the RANKL/OPG balance. Taken together, modulation of the NF-κB–RANKL/OPG axis appears to be the central mechanistic node underlying the observed biological effects, linking intestinal redox-inflammatory regulation to downstream control of osteoclast differentiation and osteoblast functional recovery. Importantly, these effects appear to involve both direct modulation of bone cell activity and indirect mechanisms related to improved intestinal homeostasis within the gut–bone axis. From a translational perspective, nutraceutical strategies that simultaneously target intestinal integrity, oxidative stress, and bone remodelling may represent promising complementary approaches to the prevention of glucocorticoid-induced osteoporosis.

Despite the mechanistic relevance of the in vitro multi-model approach adopted, the present study does not fully reproduce the endocrine, biomechanical, and microbiota-mediated complexity of bone remodelling in vivo. Furthermore, the Caco-2 basolateral fraction-based experimental design, although increasing physiological plausibility, may introduce variability related to metabolite composition and bioavailability. The limitation of this study lies in the intrinsic characteristics of the experimental model used. The Caco-2 cell monolayer is a widely validated and useful system for studying intestinal absorptive processes; however, it does not fully reproduce the complexity of the intestinal environment in vivo. Specifically, the model does not include the intestinal microbiota, a source of numerous bioactive metabolites that can influence systemic physiology and bone processes, nor the gut-associated lymphoid tissue (GALT), which modulates inflammatory and osteoimmune responses. Furthermore, because it is composed of a single epithelial cell population, the system does not fully reflect the heterogeneity of the human intestinal epithelium, which includes mucus-producing goblet cells and other specialised populations that play key roles in absorptive processes and immune signalling.

Future studies integrating in vivo models, pharmacokinetic profiling, and microbiota-dependent metabolism will be required to confirm the translational relevance of the observed synergistic effects.

## 5. Conclusions

In conclusion, the nutraceutical formulation Mix, composed of *Artemisia capillaris*, *Boswellia serrata*, *Eruca sativa*, and vitamin D3, exerts a marked osteoprotective effect by targeting multiple mechanisms involved in bone remodelling. The treatment attenuated dexamethasone-induced osteoclast activation while promoting osteoblast viability, differentiation, and mineralisation, ultimately restoring a more balanced osteogenic profile. Moreover, conditioned media from treated osteoblasts modulated osteoblast–osteoclast crosstalk, limiting osteoclast differentiation through regulation of key signalling pathways, including RANK and GSK3β.

Overall, these findings indicate that Mix enhances osteoblast function, inhibits osteoclastogenesis, and restores the RANKL/OPG balance, supporting its potential as a multi-component nutraceutical strategy for the prevention or adjunctive management of osteoporosis. However, considering the systemic complexity of osteoporosis, further in vivo studies and pharmacokinetic investigations are required to determine whether the synergistic effects observed in vitro can translate into clinically relevant benefits. In this context, future studies integrating more physiologically relevant intestinal models, including the contribution of the microbiota and immune components, will be essential to better recapitulate the in vivo environment and to further validate the translational potential of these findings.

## Figures and Tables

**Table 1:** The table summarises the chemical characterisation of the plant extracts based on HPLC analyses. *Artemisia capillaris* was characterised by quantifying scoparone and chlorogenic acid, *Boswellia serrata* by measuring total organic acids and acetylated boswellic acids, and *Eruca sativa* Mill. by determining icariin content. All methods employed external calibration curves with high linearity (R^2^ > 0.995) and reference standards to ensure accuracy and reproducibility. These data confirm the quality and compositional consistency of the extracts prior to biological evaluation.

Plant Species	Method	Component	Content (%)
*Artemisia capillaris*	HPLC	Chlorogenic acid	25 mg/g dry extract
		Scoparone	5.76 mg/g dry extract
*Boswellia serrata*	HPLC	Total organic acids	78.50%
		3-O-Acetyl-11-keto-β-boswellic acid (AKBA)	10.29%
		Acetyl-β-boswellic acid	20.58%
		Acetyl-α-boswellic acid	5.35%
*Eruca sativa* Mill.	HPLC	Icariin	10.41%

## Data Availability

The datasets generated and/or analysed during the current study are available from the corresponding author upon reasonable request, subject to reasonable conditions related to the proprietary nature of the investigated formulation.

## Abbreviations

The following abbreviations are used in this manuscript:

AKBA	3-O-Acetyl-11-keto-β-Boswellic Acid
ALP	Alkaline Phosphatase
ANOVA	Analysis of Variance
ATCC	American Type Culture Collection
BCA	Bicinchoninic Acid Assay
BMP2	Bone Morphogenetic Protein 2
Dex	Dexamethasone
DMEM	Dulbecco’s Modified Eagle Medium
ERK	Extracellular Signal-Regulated Kinase
FBS	Fetal Bovine Serum
GSK3β	Glycogen Synthase Kinase 3 Beta
HPLC	High-Performance Liquid Chromatography
IGF-1	Insulin-Like Growth Factor 1
IL	Interleukin
MAPK	Mitogen-Activated Protein Kinase
M-CSF	Macrophage Colony-Stimulating Factor
MTT	3-(4,5-Dimethylthiazol-2-yl)-2,5-Diphenyltetrazolium Bromide
NF-κB	Nuclear Factor Kappa-Light-Chain-Enhancer of Activated B Cells
NFATc1	Nuclear Factor of Activated T Cells 1
OPG	Osteoprotegerin
PBS	Phosphate-Buffered Saline
PMSF	Phenylmethylsulfonyl Fluoride
RANK	Receptor Activator of Nuclear Factor κB
RANKL	Receptor Activator of Nuclear Factor κB Ligand
ROS	Reactive Oxygen Species
Runx2	Runt-Related Transcription Factor 2
SD	Standard Deviation
TEER	Transepithelial Electrical Resistance
TJ	Tight Junction
TNF-α	Tumor Necrosis Factor Alpha
TRAP	Tartrate-Resistant Acid Phosphatase
ZO-1	Zonula Occludens-1

## Appendix A

Phenolic compounds were analysed by HPLC-PAD using a 1260 Infinity system. Extracts were dissolved, filtered, and separated on a reverse-phase C18 column. Compound identification was performed by HPLC-PAD-ESI-QTOF-MS/MS, while quantification was carried out using HPLC-DAD based on calibration curves of pure phenolic standards. When specific standards were unavailable, quantification was performed using structurally similar compounds [84].

**Table A1:** Chemical characterisation of Caco-2 basolateral fraction.

Extract	Apical Dosage	Secondary Metabolites Concentrations
*Artemisia*	40 μg/mL	0.9 μg/mL
*Eruca*	100 μg/mL	1.4 μg/mL
*Boswellia*	140 μg/mL	1.3 μg/mL
Vitamin D3	100 nM	1.1 nM

Bliss independence analysis revealed that the observed effects of the combined formulation on TRAP activity, ALP and mineralisation analysis were significantly higher than the expected values, indicating a synergistic interaction between the extracts.

**Table A2:** TRAP activity analysis. Bliss independence analysis revealed the observed effects of the combined formulation on TRAP activity. Vit D3 = Vitamin D3; Mix = *Artemisia* 40 μg/mL + *Eruca* 100 μg/mL + *Boswellia* 140 μg/mL + Vitamin D3 100 nM.

Treatment	E_exp	E_obs	Interaction
*Artemisia* + *Eruca*	0.97	1.05	Synergistic
*Artemisia* + *Boswellia*	0.91	1.02	Synergistic
*Artemisia* + Vit D3	0.79	0.94	Synergistic
*Eruca* + *Boswellia*	0.99	1.07	Synergistic
*Eruca* + Vit D3	0.98	1.04	Synergistic
*Boswellia* + Vit D3	0.94	1.00	Synergistic
Mix	1.00	1.16	Strongly synergistic

**Table A3:** ALP analysis. Bliss independence analysis revealed that the observed effects of the combined formulation on ALP levels. Vit D3 = Vitamin D3; Mix = *Artemisia* 40 μg/mL + *Eruca* 100 μg/mL + *Boswellia* 140 μg/mL + Vitamin D3 100 nM.

Treatment	E_exp	E_obs	Interaction
*Artemisia* + *Eruca*	1.03	1.18	Synergistic
*Artemisia* + *Boswellia*	1.02	1.15	Synergistic
*Artemisia* + Vit D3	0.93	1.05	Synergistic
*Eruca* + *Boswellia*	1.09	1.25	Synergistic
*Eruca* + Vit D3	1.02	1.16	Synergistic
*Boswellia* + Vit D3	0.98	1.10	Synergistic
Mix	0.99	1.54	Strongly synergistic

**Table A4:** Mineralization analysis. Bliss independence analysis revealed that the observed effects of the combined formulation on mineralization analysis. Vit D3 = Vitamin D3; Mix = *Artemisia* 40 μg/mL + *Eruca* 100 μg/mL + *Boswellia* 140 μg/mL + Vitamin D3 100 nM.

Treatment	E_exp	E_obs	Interaction
*Artemisia* + *Eruca*	0.99	1.08	Synergistic
*Artemisia* + *Boswellia*	0.92	1.06	Synergistic
*Artemisia* + Vit D3	0.86	0.98	Synergistic
*Eruca* + *Boswellia*	1.05	1.17	Synergistic
*Eruca* + Vit D3	1.01	1.11	Synergistic
*Boswellia* + Vit D3	0.97	1.04	Synergistic
Mix	0.95	1.32	Strongly synergistic
